# Establishment of bovine 3D enteroid-derived 2D monolayers

**DOI:** 10.1186/s13567-022-01033-0

**Published:** 2022-03-02

**Authors:** Kate M. Sutton, Brigid Orr, Jayne Hope, Stina R. Jensen, Lonneke Vervelde

**Affiliations:** 1 Division of Infection and Immunity, The Roslin Institute & R(D)SVS, University of Edinburgh, Easter Bush, Midlothian, EH25 9RG UK; 2 Novozymes A/S, Animal Health and Nutrition, 2800 Lyngby, Denmark

**Keywords:** Barrier function, bovine enteroids, epithelial cells, 2D enteroids, 3D enteroids, TEER

## Abstract

Three-dimensional (3D) intestinal enteroids are powerful in vitro models for studying intestinal biology. However, due to their closed structure direct access to the apical surface is impeded, limiting high-throughput applications of exogenous compounds and pathogens. In this study, we describe a method for generating confluent 2D enteroids from single-cell suspensions of enzymatically-dissociated ileum-derived bovine 3D enteroids. Confluent monolayers were first achieved using IntestiCult media but to establish a defined, cost-effective culture media, we also developed a bovine enteroid monolayer (BEM) medium. The monolayers cultured in BEM media proliferated extensively and formed confluent cell layers on both Matrigel-coated plastic plates and transwell inserts by day 3 of culture. The 2D enteroids maintained the epithelial cell lineages found in 3D enteroids and ileum tissue. In addition, the monolayers formed a functional epithelial barrier based on the presence of the adherens and tight junction proteins, E-cadherin and ZO-1, and electrical resistance across the monolayer was measured from day 3 and maintained for up to 7 days in culture. The method described here will provide a useful model to study bovine epithelial cell biology with ease of access to the apical surface of epithelial cells and has potential to investigate host–pathogen interactions and screen bioactive compounds.

## Introduction

The intestinal epithelium consists of a single layer of polarised cells that protects the host from the commensal and pathogenic bacteria while transporting nutrients and fluids from the intestinal lumen. In recent years, three-dimensional (3D) enteroids have emerged as invaluable models to study epithelial barrier function and host–pathogen interactions in veterinary species [1–11]. The 3D enteroid models can be derived from isolated stems cells or intestinal crypts that can be grown in the presence of growth factors, such as epidermal growth factor (EGF), R-spondin-1 and Noggin, and a laminin-rich extracellular matrix (ECM), e.g., Matrigel. These enteroids form organised villus-crypt structures consisting of key epithelial cell lineages resembling the intestinal epithelium in vivo (reviewed by [9–11]).

The 3D enteroids are architecturally arranged such that the apical brush borders of the epithelium face inwards while the basolateral region is in contact with the ECM [12]. A common method to access the central lumen and apical epithelium is microinjection which is labour intensive, relatively inaccurate and requires a sizeable hollow lumen which is often reduced in differentiated enteroids [13]. Alternatively, 3D enteroids can be mechanically disrupted prior to exposure to particulates, however this approach exposes both the apical and basal regions of the epithelial cells [14].

In vitro cultures of bovine immortalised or primary intestinal epithelial cultures have been widely used to study cell function and host–pathogen interactions in cattle [15–19]. However, the physiological relevance of transformed cell lines is not well understood. Furthermore epithelial cell lines mainly consist of a single cell type, enterocytes, and lack the diversity of cell types found in vivo which further contributes to their limited relevance. Deriving two-dimensional (2D) cell cultures from 3D enteroids has become an alternative approach to investigate intestinal barrier function and accessing the epithelium apical surface in pigs, humans, mice, and horses [1, 7, 20–23]. More recently bovine 3D-derived 2D colonic enteroids have been developed [24]. In this study, stem cells are maintained as colonic spheroids by continuous activation of the Wnt signalling pathway. Subsequently, 3D and 2D enteroid epithelial cell differentiation is triggered by withdrawal of Wnt3a and other niche factors [24]. Growing bovine small intestinal-derived ileum crypts in IntestiCult media adapted for mouse can sustain the stem cell niche and drive the development of key epithelial cell lineages expressed in their tissue of origin. Bovine 3D enteroids can be maintained long-term through multiple serial passages which has little influence on their growth, morphology or transcriptome, making them suitable models for the development of small intestinal 2D enteroids [2]. The 2D models allow direct access to the apical side of the epithelium through the medium [25, 26]. Thus, there is a clear need for the development of a standardized and reproducible approach to generate bovine 2D enteroids.

Here we describe a method for generating bovine ileum 3D-derived 2D enteroids that can be grown in a cost-effective, cell culture medium on Matrigel coated-plastic plates and transwell inserts. The polarised monolayers can be maintained in culture for over 1 week and consist of all epithelial cell lineages such as Paneth cells, goblet cells, enterocytes, enteroendocrine and stem cells. The formation of adherens and tight junctions leads to the generation of a functional epithelial barrier demonstrated by electrical resistance across the epithelia layer. Our approach to the generation of 2D enteroids will allow for future high-throughput applications to study the effect of food-additives, host–pathogen interactions, permeability and nutrient transport studies in cattle.

## Materials and methods

### Generation and passage of intestinal 3D enteroids

All tissues used in this study were obtained from six healthy male British Holstein–Friesian (*Bos taurus*) calves (< 6 month old) housed at the University of Edinburgh farm. Calves were subject to Schedule 1 method of cull and sections of distal ileum were obtained post-mortem and stored in Mg^2+^ and Ca^2+^ free Hank’s Buffer Saline Solution (HBSS, Life Technologies, Paisley, UK) on ice until use. Crypts were isolated as previously described [2]. For 3D enteroid cultures, 10^3^ crypts were resuspended in Matrigel^®^-GFR (Corning, Loughborough, UK) and seeded in 50 µL centric domes in 24-well plates (Corning). After a 10 min polymerisation period at 37 °C, 5% CO_2_, 650 µL of complete IntestiCult Mouse medium (STEMCELL Technologies UK Ltd) was added per well as described previously [1]. This 3D medium was supplemented with 10 μM SB202190 (Enzo Life Sciences, Europe), 10 μM Y-27623 dihydrochloride, 500 nM LY2157299 (Cambridge BioScience, Cambridge, UK) and 25 µg/mL gentamicin (Sigma-Aldrich, Gillingham, UK). Half of the culture media was replaced every two days to retain intrinsic growth factors produced by the 3D enteroids and was found to have no detrimental effect on 3D enteroid growth and morphology.

For weekly passage of the enteroids, culture medium was removed and replaced with 1 mL of ice-cold wash media; Advanced DMEM/F12 with 1 × B27 Plus supplement (GIBCO, Paisley, UK). Matrigel was dissociated by repeated pipetting with 1000 µL tip and transferred to a fresh 15 mL conical tube. At least four wells were pooled from the same animal at this stage. Enteroids were placed on ice for 5 min to settle by gravity. The supernatant was aspirated and discarded and the enteroids were resuspended in 1 mL of ice-cold wash media and placed in 1.5 mL Eppendorf tube. The enteroids were mechanically dissociated by repeated pipetting ~35 times with a 200 µL tip and retained on ice. The level of enteroid dissociation was examined using a microscope by placing 10 µL of cells on a 24-well plate. The dissociated enteroid fragments were counted to seed approximately 1000 fragments per 50 µL. The 50 µL was then mixed with 150 µL of Matrigel and 50 µL centric domes were seeded on 24-well plates then allowed to polymerise for 10 min at 37 °C, 5% CO_2_. Each well received 650 µL of complete IntestiCult Mouse media. Half of the culture media was replaced every two days.

### Generation of 2D enteroids

After three passages and on day 5 of culture, bovine 3D enteroids, derived from 6 individual calves, were recovered from the Matrigel domes by gentle pipetting using 200 µL tip with 1 mL of ice cold wash media. Each well was further washed with 1 mL of wash media. Enteroids were further mechanically disrupted by vigorous pipetting ~35 times using a 200 µL tip and transferred to a fresh 15 mL conical tube. The disrupted enteroids were pelleted at 300 *g* for 4 min, supernatant was removed, and cells were resuspended in 1 mL of TrypLE™ Express (ThermoFischer Scientific, Paisley, UK. TFS) and transferred to a 24-well plate (Corning). The disrupted enteroids were digested for 10 min at 37 °C, 5% CO_2_. To further encourage enteroid breakdown into single cells, cells were mechanically disrupted ~40 times using a 200 µL tip. The enzymatic reaction was quenched by the addition of four volumes of pre-warmed DMEM supplemented with 10% FBS (GIBCO). The cells were filtered twice through a 40 µm cell strainer (VWR) to obtain a single cell suspension. Cells were pelleted for 5 min at 300 *g*. To examine the ability to form a confluent layer, the single cells were seeded at 2.5 × 10^4^ cells/well on 2% (v/v) Matrigel coated 96-well flat plates (Corning) or the apical region of 24-well transwells (0.33 cm^2^). Cells were cultured in complete IntestiCult Mouse media as outlined above, supplemented with 20% (v/v) FBS or bovine enteroid monolayer (BEM) media consisting of the components outlined in Table 1. BEM media was supplemented with 20% or 1% (v/v) FBS for initial growth analysis and 1% FBS was used thereafter. The plate coating procedure involved incubation of 2% (v/v) Matrigel in DMEM/F12/1 × B27 Plus supplement medium at 37 °C, 5% CO_2_ for 1 h after which the liquid was removed. For 96-well flat plates, cells were cultured in 200 µL of media and for transwells, 200 µL of media was added to apical chamber while 500 µL of media was added to the basal chamber. Forty-eight hours post-seeding, the media in the apical and basal chambers were replaced and refreshed every two days thereafter. After 3 days of culture the cells reached confluence and were used for further experiments.

**Table 1:** **Composition of bovine enteroid monolayer (BEM) media**.

Reagent	Concentration	Supplier
Advanced DMEM/F12	1×	ThermoFisher Scientific (TFS)
l-glutamine	2 mM	TFS
HEPES	10 mM	TFS
Penicillin/Streptomycin	50 U/mL	TFS
B27 supplement	1×	TFS
N2 supplement	1×	TFS
EGF (human)	50 ng/mL	TFS
LDN 193189	100 nM	Cambridge Bioscience
R-Spondin 1 (human)	100 ng/mL	R&D Systems
Noggin (human)	50 ng/mL	R&D Systems
CHIR99021	10 µM	Stratech Scientific
Y27632	10 µM	Cambridge BioScience
SB202190	10 µM	Enzo Life Sciences
LY2157299	500 nM	Cambridge BioScience
Nicotinamide	10 mM	Sigma-Aldrich
FBS	1%	GIBCO

### RNA isolation, reverse transcription quantitative polymerase chain reaction (RT-qPCR)

For RNA isolation, on day 5 of culture four wells of 3D enteroids generated from four individual calves were collected from Matrigel domes as outlined above. The 2D enteroids derived from the same 3D enteroids mentioned above were seeded at 2.5 × 10^4^ cells/well on 2% (v/v) Matrigel coated 96-well plates with three wells per calf in BEM media for 5 days. The cells were recovered by gentle pipetting using 200 µL tip with 0.2 mL of ice cold wash media and placed in a 1.5 mL Eppendorf. After 3D and 2D enteroids were mechanically dissociated in 1.5 mL Eppendorf, the cells were pelleted at 300 *g* for 5 min and resuspend in RLT buffer (QIAGEN, Crawley, UK) supplemented with β-mercaptoethanol (1:100). Ileum tissue (30 mg) was homogenised using TissueLyser II (QAIGEN) and steel beads in RLT buffer supplemented with β-mercaptoethanol (1:100). Total RNA was extracted using RNeasy PLUS kit (QIAGEN) consisting of a genomic DNA column eliminator according to manufacturer’s instructions, and quantified spectrophotometrically. Complementary DNA (cDNA) samples were prepared using 0.5 μg total RNA per sample, 10 nM random hexamers and Oligo dT_20_ and SuperScript III Reverse Transcription Kit (Invitrogen, Paisley, UK) according to manufacturer’s instructions. For RT-qPCR, 1:5 dilution of cDNA was mixed with 10 µL of ABI TaqMan Gene Expression Master Mix (Applied Biosystems, TFS), 1 µL of 20X EvaGreen (Biotum, VWR, Lutterworth, UK) and 1.15 µm of primers against bovine Lgr5 (F:ACTTTCCAGCAGTTGTTCAGC; R:GAATAGACGACAGGCGGTTG, NM_001192520.3), Chromogranin A (F:GGGACACTGAGGTGATGAAG; R:GTCGCAGGATTGAGAGGAT, NM_181005.2), Mucin 2 (F:ATGGCACCTACCCGTTCAC; R:AATCTCGCTCTTCACCTGGA, XM_024987595.1), Lysozyme C (F:TTCCTTTCTGTTGCTGTCCA; R: AGCCATCCAGTCCAAGTTTC, NM_001080339). Data was normalised against two bovine reference genes, actin beta (F:CCAACCGTGAGAAGATGACC; R:CCAGAGGCATACAGGGACAG, NM_173979.3) and spliceosome-associated factor 1 (F:ATGAAGAAGCTGGACGAGGA R:GGAGGGATCAGAAGGGAGAC, NM_001193086.2). The stability of the reference genes were evaluated by NormFinder using GeneEx program (V6) [27]. Data are expressed in terms of the Corrected 40-cycle threshold (Ct) value, which is normalised using the Ct values of actin beta and spliceosome-associated factor 1 for each sample using the formula; Ct + (N′t-C′t)*(S/S′) where N′t is the mean Ct value for the reference genes in each cell culture or tissue, C′t is the mean value for reference genes in the sample and the S and S′ are the slopes of regression of the standard plots for both reference genes and the gene of interest, respectively. Normalised Ct values were corrected against the negative association of the log concentration of cDNA detected using the formula 40—normalised Ct value of the gene of interest.

### Immunofluorescence staining and imaging

To examine cell proliferation and immunostaining of the 2D enteroids, cells were seeded at 2.5 × 10^4^ cells/well on 2% (v/v) Matrigel coated 8 chamber glass slides (TFS) or on 24-well transwell inserts (0.33 cm^2^). On day 5 of culture, proliferating cells were visualised by adding 5-ethynyl-2′-deoxyuridine (EdU, 10 µM) to the cultures for 2 h at 37 °C, 5% CO_2_ and stained according to manufacturer’s instructions (Base Click, Munich, Germany). The cells were fixed apically and basally using 4% (v/v) paraformaldehyde in PBS (Mg^2+^ and Ca^2+^ free) for 10 min at RT. Cells were permeabilised with 0.5% (v/v) Triton X-100 in PBS for 10 min at RT, washed and blocked for 20 min at RT with 20% (v/v) goat serum (Chondrex) in PBS. Primary and secondary antibodies were diluted in 0.1% (v/v) Triton X-100 in PBS and incubated overnight at RT. Washing steps were performed by applying PBS three times to both the apical and basolateral chambers for 5 min. Primary antibodies used were mouse anti-human E-cadherin (Clone 36, 1:50, IgG2a, BD Biosciences), mouse anti-human ZO-1 (Clone 1A12, 1:100, IgG1, Invitrogen), mouse anti-villin (Clone 1D2C3, 1:100, IgG1, Santa Cruz), mouse anti-cytokeratin 18 (Clone C-04, 1:100, IgG1, Abcam), or the lectin, Ulex Europaeus Agglutinin I (UEA-1)-biotin (1:300, VectorLabs). Secondary antibodies included goat anti-mouse IgG1/IgG2a Alexa Fluor™-488, -594 or -568 (1:300) or streptavidin-Alexa Fluor^TM^594 (1:100, TFS). Cells were counterstained with Hoechst 33458 (Sigma-Aldrich). Transwell membranes were cut from the inserts using a scalpel and placed with cells facing upwards on microscopic slides with reaction wells (11 mm Ø, Marienfeld, Germany). All inserts were mounted using ProLong Diamond Antifade Mountant (TFS). Differential interference contrast (DIC) and brightfield images were captured on Axiovert 25 microscope (Zeiss). Fluorescent images and Z stacks were captured using an inverted LSM880 (Zeiss) using 40× or 63× oil lens. Data was analysed using IMARIS (V9.6) and ZEN blue (Zeiss).

### Trans-epithelial electrical resistance (TEER)

TEER was measured using an epithelial volt-ohmmeter (EVOM2, World Precision Instruments) and a chopstick electrode (STX2). Each transwell insert was measured three times (once in each pore) and average values were corrected against the average background TEER and surface area as follows; TEER Ωcm^2^ = (R_cell layer_ − R_blank_) × A. R_cell layer_ is the resistance (R) of the cell monolayer in coated wells with cells; R_blank_ is the resistance of the coated well without cells, and A is the surface area of the well (0.33 cm^2^).

## Results

### Generation of bovine 3D-derived 2D enteroids

**Figure 1:**
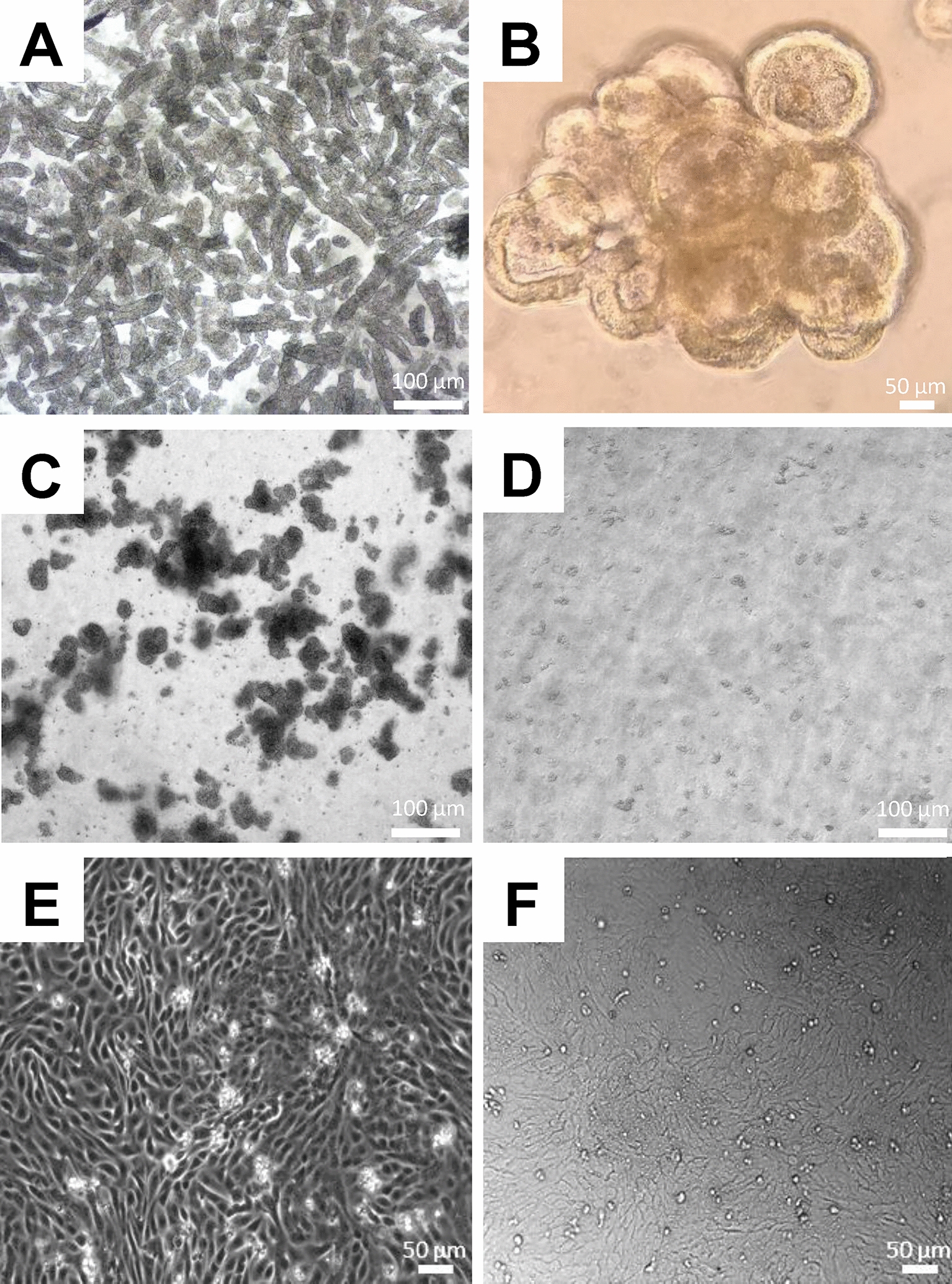
**Generation of bovine 3D-derived 2D enteroids.** Representative brightfield images of (**A**) crypts directly after isolation from distal ileum of a 6-month-old calf, **B** Matrigel-embedded crypts form villus-crypts structures by day 7 of culture. Data is representative of six independent experiments using six individual calves. Enteroids were passaged three times and on day 5 of culture 3D enteroids were **C** mechanically disrupted by vigorous pipetting and **D** enzymatically dissociated into single cells using TrypLE Express. **E** Representative DIC image of the morphology of 2D enteroid cultured for 3 days at 2.5 × 10^4^ cells/well on 2% Matrigel coated 96-well plate in complete IntestiCult mouse media supplemented with 20% FBS. **F** Representative brightfield image of 2D enteroids at day 10 of culture. Data is representative of three independent experiments using three individual calves.

The biggest drawback of the current bovine 3D enteroids is the labour intensive approach to gain access to the apical surface of the epithelium. To circumvent this, we set out to generate bovine 3D-derived 2D enteroids while maintaining the cellular heterogeneity of the in vivo intestine to ensure physiological relevance. Bovine 3D enteroids were generated from crypts isolated from the distal ileum tissue of 6-month-old calves and cultured as described [2] (Figure 1A). To exclude leukocytes and fibroblasts, the 3D enteroids were passaged at least three times prior to generating 2D enteroids (Figure 1B). After the 3rd passage, 5-day-old bovine 3D enteroids were mechanically disrupted and enzymatically dissociated into single cells (Figures 1C and D). Bovine cells were cultured at the same seeding density as porcine 2D enteroids and in complete IntestiCult media supplemented with 20% FBS on Matrigel coated 96-well plates [2]. Three days after seeding, confluent cell layers were observed with a cobblestone morphology (Figure 1E) which could be maintained for a further 7 days (Figure 1F). Bovine 3D enteroids dissociated into single cells and seeded at 2.5 × 10^4^ cells/well in IntestiCult media supplemented with 20% FBS can form a confluent 2D enteroid monolayer on Matrigel coated 96-well plates.

### Confluent 2D enteroids can be established using BEM medium

Next, we explored the ability to grow confluent 2D enteroids using growth medium adapted from murine, porcine and bovine studies to produce a cost-effective medium with defined culture components [1, 2, 21]. This bovine 2D enteroid monolayer or BEM medium consisted of the Rho kinase-, p38 MAP kinase- and TGFβR-inhibitors in the same concentrations used in the bovine 3D enteroid cultures [2]. The BEM media also consisted of growth factors, Noggin, R-spondin-1, EGF, and CHIR99021 along with N2 and B27 supplements. The ability of the cultures seeded at 2.5 × 10^4^ cells/well on Matrigel-coated 96-well plates to form a confluent monolayer in BEM media supplemented with 20% or 1% FBS were examined by microscopy.

**Figure 2:**
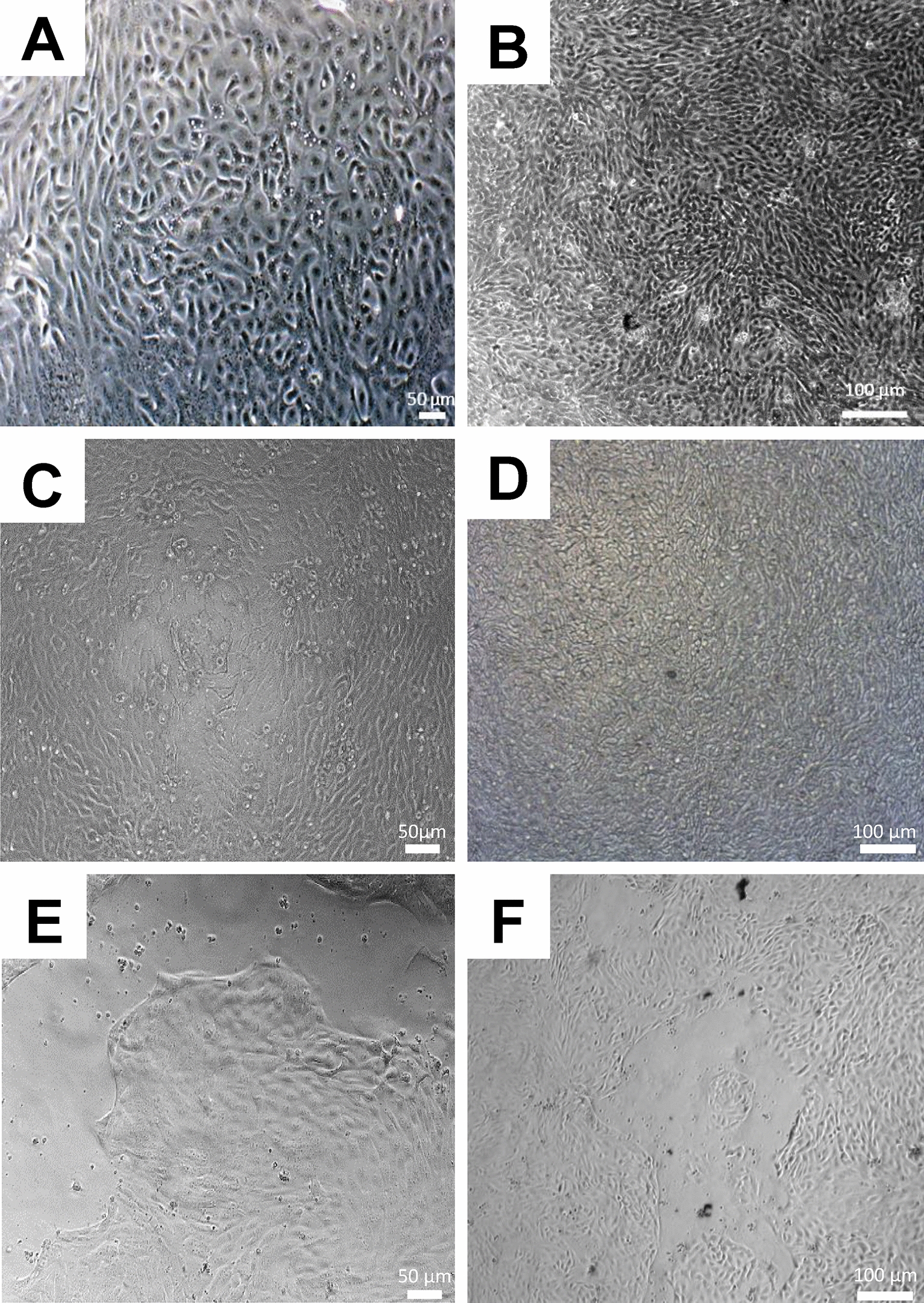
**Formation of confluent 2D enteroids cultured in BEM media.** Representative DIC images of 3-day-old 2D enteroids in BEM media supplemented with **A** 20% or **B** 1% FBS. Representative brighfield images of 10-day-old 2D enteroids in BEM media supplemented with **C** 20% or **D** 1% FBS. Representative brighfield images of 2D enteroids in BEM media without FBS on **E** day 3 and **F** day 4 of culture. Cells were seeded at 2.5 × 10^4^ cells/well on Matrigel coated 96-well plate. Data is representative of three independent experiments from three individual calves.

At day 3 post-seeding, confluent cell layers with a cobblestone morphology, characteristic of epithelial cell cultures, were observed for cells cultured in BEM media supplemented with 20% or 1% FBS (Figures 2A and B). The 2D enteroids could be cultured for up to 10 days post-seeding (Figures 2C and D). This indicates that low levels of FBS can support bovine epithelial cell growth. We next analysed the formation of 2D enteroid monolayers using BEM media with no FBS and found that the cells did not form a confluent monolayer on days 3 or 4 of culture (Figures 2E and F), but a monolayer was observed by day 5 of culture (data not shown, similar to Figures 2A and B). This demonstrates the 2D enteroids can be generated in BEM media without FBS, but confluency is achieved at a much slower rate compared to cells grown in the presence of FBS. Using BEM media, 2D enteroids form a confluent cell layer similar to cells cultured in complete IntestiCult mouse media. BEM media supplemented with 1% FBS was used in further studies.

### Representation of epithelial lineages in bovine 2D enteroids

**Figure 3:**
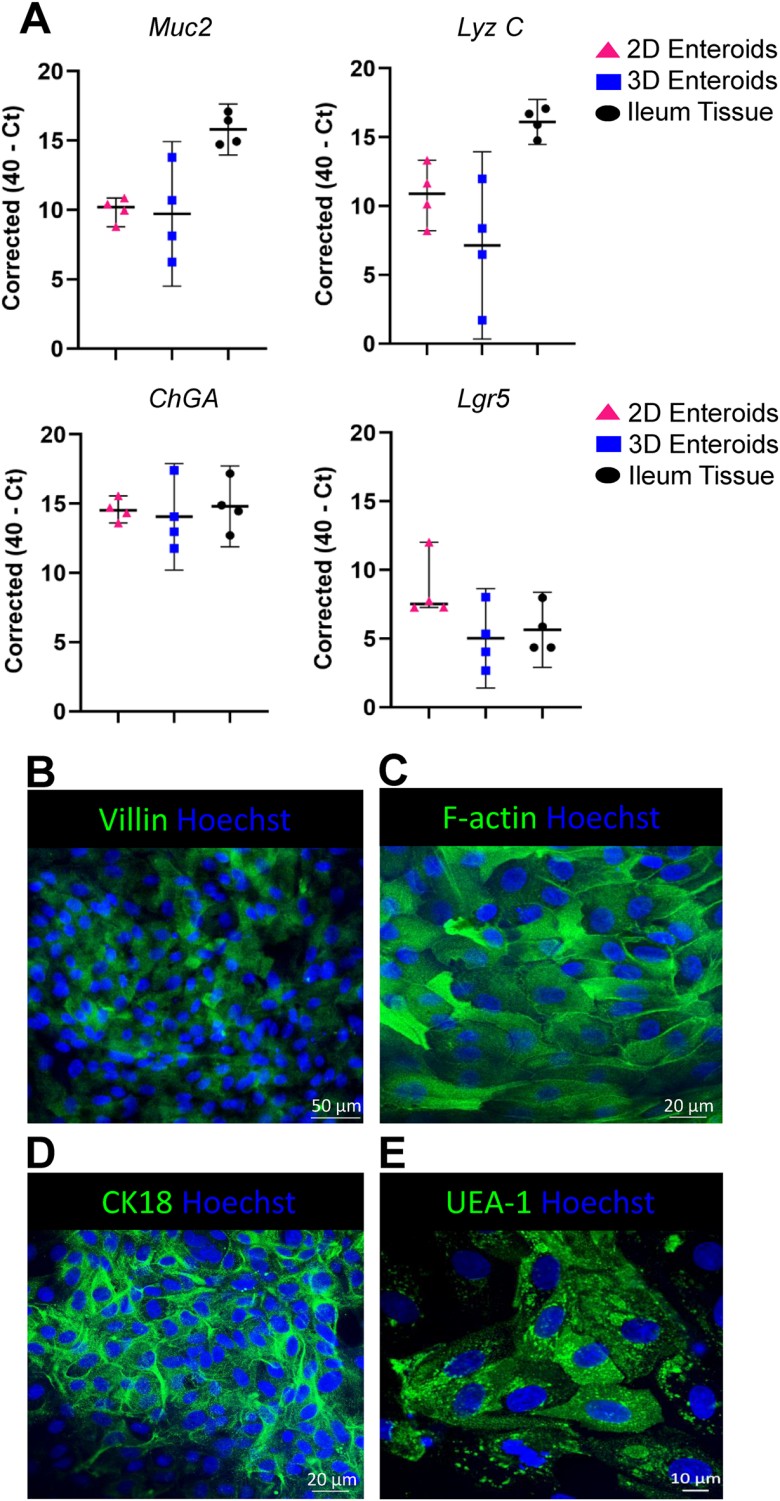
**Bovine 2D enteroids consist of multi-epithelial cell lineages**. **A** Reverse transcriptase-qPCR gene expression profiles of epithelial cell markers; Mucin2 (*Muc2*) for Goblet cells, Lysozyme C (*Lyz C*) for Paneth cells, Chromogranin A (*CHGA*) for Enteroendocrine cells and Leucine-rich Repeat containing G-protein Receptor 5, (*Lgr5*) for stem cells in 2D enteroid cultures grown on Matrigel coated 96-well plates in BEM/1% FBS media for 5 days, 3D enteroids cultured in IntestiCult media for 5 days and ileum tissue of 6-month old calves of enteroid origin. Data represents the median and error bars ± 95% confidence interval of four independent experiments. Representative confocal images of 5-day-old 2D enteroids demonstrating the presence of **B** villin (green) expression in enterocytes and **C** F-actin (green) filaments, **D** the bovine epithelial cell marker, cytokeratin 18 (green), and **E** UEA-1 (green) binding was observed in numerous cells. Nuclei are visualised by Hoechst staining (blue). The 2D enteroids were seeded at 2.5 × 10^4^ cells/well on Matrigel coated 8-well-chamber glass slides in BEM/1% FBS media for 5 days. Data is representative of five independent experiments of five individual calves.

Next we analysed the gene expression profiles of known epithelial cell markers in the ileum tissue of 3D enteroid origin and in the 3D and 2D enteroid cultures by RT-qPCR. Five-day-old 2D enteroids cultured in BEM media expressed the goblet cell associated gene, Mucin 2 (*Muc2*). *Muc2* mRNA expression levels in the 2D enteroids were less variable compared to the 3D enteroids which were cultured in IntestiCult media (Figure 3A). In addition, the Paneth cell gene, Lysozyme C (*Lyz C*), mRNA expression levels was more consistent across the four independent 2D enteroid cultures compared to the 3D enteroids. The mRNA expression levels of the enteroendocrine cell gene, Chromogranin A, (*CHGA*), was similar across the enteroid cultures and in the ileum tissues. The stem cell gene, Leucine-Rich Repeat containing G-protein Receptor 5, (*Lgr5*) was expressed at slightly higher levels in 2D enteroids compared to the 3D enteroids and ileum tissue (Figure 3A). Our data indicates that bovine 3D-derived 2D enteroids grown in BEM media sustained the epithelial cell heterogeneity found in the 3D enteroids and ileum tissue of origin.

Immunofluorescence analysis of the 2D enteroids demonstrates the expression of villin, which is an actin-binding protein expressed in differentiated enterocytes (Figure 3B), and staining with phalloidin, which binds to F-actin filaments (Figure 3C). Cytokeratin 18 was found to be expressed in most cells (Figure 3D). In addition, we examined UEA-1 binding in 2D enteroids and found it binds to the majority of cells (Figure 3E). Overall, BEM media can support the maintenance of stem cells and differentiation of key epithelial cell lineages in 2D enteroids.

### Bovine 2D enteroids form a tight barrier

**Figure 4:**
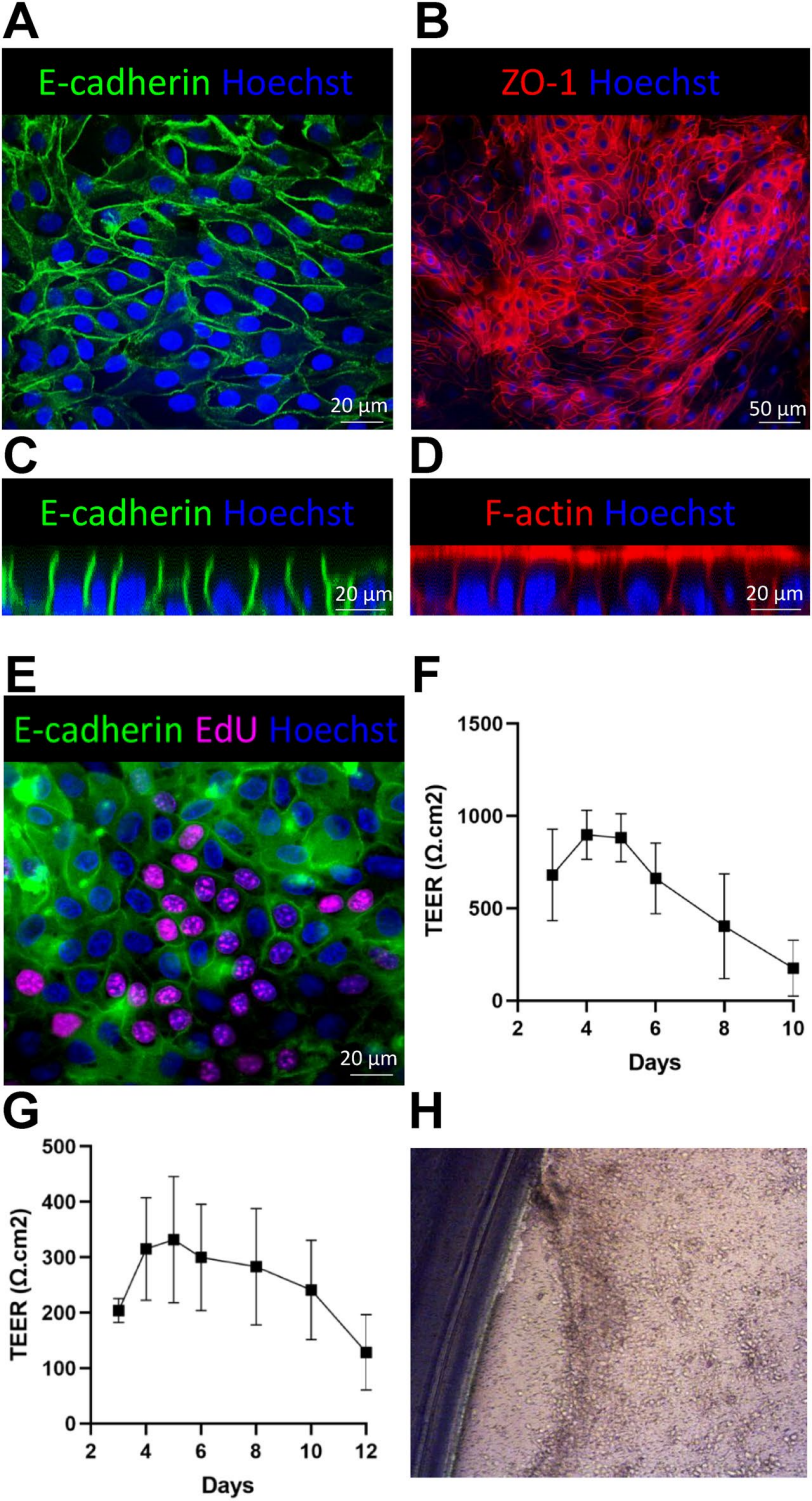
**Bovine 2D enteroid form tight junctions and a functional epithelial barrier. A** Representative confocal images of the adherens junction protein, E-cadherin (green) and **B** 3X3 tile image demonstrating uniformed expression of the tight junction protein ZO-1 (red) in 2D enteroids seeded at 2.5 × 10^4^ cells/well on Matrigel coated transwell inserts in BEM/1% FBS media. **C** Z-stack modeling indicate the expression of E-cadherin (green) between the cells and **D** strong apical expression of F-actin (red) in the 2D enteroids. **E** EdU (magenta) uptake by E-cadherin^+^ (green) proliferating cells in 2D enteroids. Nuclei are visualised by Hoechst staining (blue). Cells were imaged on Matrigel coated transwell inserts. **F** Short-term, high TEER can be achieved by seeding 2.5 × 10^5^ cells/well and **G** a lower more stable TEER can be reached using 2.5 × 10^4^ cells/well. Data is the median of 5 (**F**) or 12 (**E**) independent experiments from 5 to 6 calves. Error bars ± 95% Confidence Interval. **H** Representative brightfield image of the destablistion of the 2D enteroids leading to a drop in TEER on day 12 of culture on transwell inserts.

Epithelial monolayers grown on transwell inserts can be used to investigate epithelial barrier integrity by measuring transepithelial electrical resistance (TEER). Adherens junctions consist of the transmembrane protein, E-cadherin junctional protein, which was strongly expressed between the cells cultured on transwells (Figure 4A). Zonula occludens-1 (ZO-1) also known as Tight Junction Protein-1, was expressed between the cells in the 2D enteroids and together the expression of these proteins suggests the formation of epithelial cell impudence (Figure 4B). We next analysed the polarisation of the 2D enteroids using Z-stack modelling and found strong expression for E-cadherin between the cells and apical expression of F-actin (Figures 4C and D). In addition, numerous E-cadherin^+^ EdU^+^ cells were observed in 2D enteroids at day 5 of culture indicating that BEM media can support tight junction formation and cell proliferation on transwells (Figure 4E).

To determine the optimal cell seeding density to obtain a stable TEER, transwells were seeded in BEM media at 2.5 × 10^5^ or 2.5 × 10^4^ cells/well. A seeding density of 2.5 × 10^5^ cells/well displayed TEER of 680 Ω cm^2^ from day 3 that increased on day 4 and 5 which decreased thereafter (Figure 4F). In contrast, cells seeded at 2.5 × 10^4^ cells/well yielded a lower but more stable, long lasting TEER, reaching ~200 Ω cm^2^ from day 3 and maintaining a TEER of ~280–315 Ω cm^2^ until day 8 and 10 (Figure 4G). Microscopic analysis of the transwell inserts on the day where TEER value reached the same levels as coated wells with no cells (~100 Ω cm^2^), indicated that the cell layer retracted from side of the insert, possible due to the breakdown of Matrigel or overgrowth of the cell layer (Figure 4H). The immunostaining of tight junction proteins and the TEER analysis demonstrates the formation of a functional epithelial barrier in bovine 2D enteroids.

## Discussion

The development of bovine 3D enteroids represented a novel physiologically relevant model of the bovine small intestine [2]. Transcriptomic analysis of bovine 3D enteroids demonstrated that at least one passage reduced the leukocyte and fibroblast related gene signatures [2]. To reduce these cell types in our 3D enteroids and hence 2D enteroids, cells were passaged at least three times prior to the generation of the monolayers. Cell seeding density is critical for the generation and long-term maintenance of confluent epithelial monolayers. Porcine 3D-derived 2D enteroids were found to optimally grow at 2.5 × 10^4^ cells/well in 96-well format and in media supplemented with 20% FBS [1]. Using this previously optimised approach, single cells from dissociated bovine 3D enteroids were seeded at the 2.5 × 10^4^ cells/well in 96-well format and cultured in bovine 3D IntestiCult media supplemented with 20% FBS. After 3 days, confluent cell layers were achieved (Figure 1).

The composition of culture growth medium for epithelial cells requires a number of growth factors to sustain the stem cell niche [21]. Bovine 3D enteroids are grown in IntestiCult mouse media supplemented with Rho kinase-, p38 MAP kinase- and TGFβR-inhibitors that encourage epithelial proliferation and inhibits epithelial-mesenchymal cell transition [2, 26, 28]. However, the exact composition of the IntestiCult media is undisclosed and the protein content varies from batch to batch. Crucial vitamins or hormones can be provided in the cell culture medium by FBS but its components are undefined. Alternatively, the addition of N2 and B27 supplements can be used to replace or reduce FBS in culture media [28]. Growth factors are also an important component of the cell growth medium. Noggin, R-spondin-1, EGF, and CHIR99021 collectively inhibit the bone-morphogenetic protein signalling, activate the Wnt pathway and drive epithelial cell proliferation and differentiation [12, 26, 29]. These signalling pathways work in concert to sustain epithelial cell growth and maintenance [21, 26, 29]. Culturing bovine 2D enteroids in Advanced DMEM/F12 consisting of a combination of these inhibitors, supplements and growth factors, a media we named BEM, with either 20% or 1% FBS led to the formation of confluent cell layer within 3 days post-seeding on 96-well plates and 24-well transwell inserts (Figures 2 and 4). It should be noted that bovine 3D enteroids growth could not be sustained in the same media conditions (data not shown). The ability to culture bovine 2D enteroids in BEM media reduces the culture costs and will allow access to the technology more readily worldwide.

The small intestinal epithelium and their derived 3D enteroids consists of actively proliferating Lgr5+ intestinal stem cells from which differentiated epithelial cell types are derived. These differentiated cells include absorptive enterocytes, multiple secretory cell types, Paneth cells, goblet cells, enteroendocrine cells, and the antigen-sampling M cells that overlay the follicle-associated epithelium (FAE) of the Peyer’s patches [12, 30]. Expression of markers for Paneth cells (*Lyz C*), goblet cells (*Muc2*), enteroendocrine cells (*ChGA*) and stem cells (*Lgr5*) were stable across four individual 2D enteroids cultured in BEM media. Interestingly, the expression levels of these epithelial markers were more consistent in the 2D enteroids compared to the 3D enteroids which may be attributed to differences in media composition (Figure 3A). Cytokeratin 18 is an epithelial cell marker in bovine primary epithelial cell cultures or transformed epithelial cell lines [16, 17] and recently shown to be expressed in bovine 2D colonic enteroids [24]. In vivo, cytokeratin 18 expression has been suggested as a marker for bovine and porcine M cells in the small intestinal FAE [31, 32]. The α-linked fucose binding lectin, UEA-1, has also been shown to bind to M cells in mouse intestinal FAE [33]. Bovine 3D enteroids do not express murine M cell-specific gene signatures [2] whereas M cells can be induced in murine and human 3D enteroids after treatment with the cytokine, RANKL [34, 35]. We observed uniform expression of cytokeratin 18 and binding of UEA-1 in numerous cells in the 2D enteroid culture suggesting they are not specific bovine M cell markers (Figures 3D and E). Although our analysis does not prove the existence of M cells in bovine 2D enteroids, the ability to induce M cell development in bovine enteroids by RANKL treatment is required in future studies to better understand their biology in cattle.

Bovine intestinal 3D enteroids can be cultured and passaged for several months maintaining similar expression profiles to freshly isolated intestinal tissue [2]. Due to the natural structure of mammalian 3D enteroids with the apical border of the epithelial cells facing a central, closed lumen, the surface that typically interacts with microorganisms and nutrients is not directly accessible and application of particulates to the lumen is cumbersome [13]. More recently apical-out, reversed 3D enteroids, have been described for pig, chicken, mouse and sheep [6, 7, 36, 37]. These inside-out models allow easy access to the apical surface through the medium making modelling host–pathogen infections in vitro significantly more practical. The only drawback of mammalian apical-out enteroids is their limited life-span once their orientation has been altered and ~20% enteroids fail to reverse their polarity [11, 36]. Murine 2D enteroids can be passaged at least four times [29]. The limitation of this current study is the lack of research on the ability to expand and passage the 2D enteroids, like classical cell lines. Our bovine 2D enteroids expressed *Lgr5* at mRNA level and contained numerous Edu^+^ proliferating cells suggesting their high self-renewal properties (Figures 3 and 4). Future research into the ability to cryopreserved and resurrect 2D enteroids that can retain their functionality similar to their tissue of origin would complement high-throughput bovine 2D enteroid platforms.

Barrier integrity requires the formation of tight junctions, adherens junctions and desmosomes [38, 39]. We confirmed the epithelium cell–cell contacts by E-cadherin and ZO-1 expression and determined the polarised nature of the 2D enteroids by Z-stack modelling (Figure 4). The transwell system allows for TEER measurement to evaluate epithelial monolayer growth and tight junction integrity, both indicators of epithelia barrier function [40]. Our 2D enteroids grown in BEM media at 2.5 × 10^5^ cells/well, generated a high TEER for two days after which it rapidly decreased. In contrast, cells seeded at a 2.5 × 10^4^ cells/well produce a lower TEER from day 3 that was sustained over a 7-day period. The TEER values in this study were similar but more stable than the TEER of a previous study using bovine colonic 3D-derived 2D enteroids. This study reported TEER of ~300 Ω cm^2^ but measurements were only analysed for a 24 h period [24]. Although our TEER is lower than that reported for porcine [1], its similar to the human 2D enteroids generated from proximal and distal fetal intestinal 3D organoids [20] and is higher than the physiological range reported for murine small intestine and 2D enteroids (40–100 Ω cm^2^) [41, 42].

Our bovine 2D enteroids reflect the cellular composition, polarity and barrier function of their in vivo counterparts [1]. The 2D enteroids further provide a practical model in which bacteria, parasites and viruses can be applied to the cell culture medium which exposes pathogens to epithelial apical surfaces. Human 2D enteroid cultures have been shown to provide excellent models for practical applications [21, 24] and in accordance our bovine model may support the screening of dietary and microbial metabolites, study host–pathogen and host-microbiome interactions, and measure transport across the intestinal barrier in bovine species. The current model of culturing 2D enteroids on transwell inserts can also be adjusted to investigate cross-talk between the epithelial monolayer and mesenchymal and immune cells through co-culture studies. Our data suggest that the 2D enteroid cultures represent a novel and practical in vitro model with a broad range of applications.

## Data Availability

The data that support the findings of this study are available on request from the corresponding authors.

## Abbreviations

BEM media: bovine enteroid monolayer media
CHGA: Chromogranin A
cDNA: complementary DNA
EGF: epidermal growth factor
EdU: 5-Ethynyl-2′-deoxyuridine
ECM: extracellular matrix
FBS: foetal bovine serum
FAE: follicle-associated epithelium
Lgr5: Leucine-rich containing G-coupled receptor 5
PCR: polymerase chain reaction
RT: room temperature
3D: three dimensional
TEER: trans-epithelial electrical resistance
2D: two dimensional
v/v: volume/volume
ZO-1: Zonula occludens-1

## References

[1] van der Hee B, Loonen LMP, Taverne N, Taverne-Thiele JJ, Smidt H, Wells JM (2018). Optimized procedures for generating an enhanced, near physiological 2D culture system from porcine intestinal organoids. Stem Cell Res.

[2] Hamilton CA, Young R, Jayaraman S, Sehgal A, Paxton E, Thomson S, Katzer F, Hope J, Innes E, Morrison LJ, Mabbott NA (2018). Development of in vitro enteroids derived from bovine small intestinal crypts. Vet Res.

[3] Stewart AS, Freund JM, Gonzalez LM (2018). Advanced three-dimensional culture of equine intestinal epithelial stem cells. Equine Vet J.

[4] Chandra L, Borcherding DC, Kingsbury D, Atherly T, Ambrosini YM, Bourgois-Mochel A, Yuan W, Kimber M, Qi Y, Wang Q, Wannemuehler M, Ellinwood NM, Snella E, Martin M, Skala M, Meyerholz D, Estes M, Fernandez-Zapico ME, Jergens AE, Mochel JP, Allenspach K (2019). Derivation of adult canine intestinal organoids for translational research in gastroenterology. BMC Biol.

[5] Li L, Fu F, Guo S, Wang H, He X, Xue M, Yin L, Feng L, Liu P (2019). Porcine intestinal enteroids: a new model for studying enteric coronavirus porcine epidemic diarrhea virus infection and the host innate response. J Virol.

[6] Nash TJ, Morris KM, Mabbott NA, Vervelde L (2021). Inside-out chicken enteroids with leukocyte component as a model to study host-pathogen interactions. Commun Biol.

[7] Hellman S (2021). Generation of equine enteroids and enteroid-derived 2D monolayers that are responsive to microbial mimics. Vet Res.

[8] Kar SK, Wells JM, Ellen ED, te Pas MFW, Madsen O, Groenen MAM, Woelders H (2021). Organoids: a promising new in vitro platform in livestock and veterinary research. Vet Res.

[9] Pain B (2021). Organoids in domestic animals: with which stem cells?. Vet Res.

[10] Beaumont M, Blanc F, Cherbuy C, Egidy G, Giuffra E, Lacroix-Lamandé S, Wiedemann A (2021). Intestinal organoids in farm animals. Vet Res.

[11] Seeger B (2020). Farm animal-derived models of the intestinal epithelium: recent advances and future applications of intestinal organoids. ATLA.

[12] Sato T, Vries RG, Snippert HJ, van de Wetering M, Barker N, Stange DE, van Es JH, Abo A, Kujala P, Peters PJ, Clevers H (2009). Single Lgr5 stem cells build crypt-villus structures in vitro without a mesenchymal niche. Nature.

[13] Wilson SS, Tocchi A, Holly MK, Parks WC, Smith JG (2015). A small intestinal organoid model of non-invasive enteric pathogen–epithelial cell interactions. Mucosal Immunol.

[14] Saxena K, Blutt SE, Ettayebi K, Zeng X-L, Broughman JR, Crawford SE, Karandikar UC, Sastri NP, Conner ME, Opekun AR (2016). Human intestinal enteroids: a new model to study human rotavirus infection, host restriction, and pathophysiology. J Virol.

[15] Kuroda K, Kiyono T, Isogai E, Masuda M, Narita M, Okuno K, Koyanagi Y, Fukuda T (2015) Immortalization of fetal bovine colon epithelial cells by expression of human cyclin D1, mutant cyclin dependent kinase 4, and telomerase reverse transcriptase: an in vitro model for bacterial infection. PLoS One 10:e0143473 10.1371/journal.pone.0143473 PMC4666463 26624883

[16] Miyazawa K, Hondo T, Kanaya T, Tanaka S, Takakura I, Itani W, Rose MT, Kitazawa H, Yamaguchi T, Aso H (2010). Characterization of newly established bovine intestinal epithelial cell line. Histochem Cell Biol.

[17] Kaushik RS, Begg AA, Wilson HL, Aich P, Abrahamsen MS, Potter A, Babiuk LA, Griebel P (2008). Establishment of fetal bovine intestinal epithelial cell cultures susceptible to bovine rotavirus infection. J Virol Methods.

[18] Birkner S, Weber S, Dohle A, Schmahl G, Föllmann W (2004). Growth and characterisation of primary bovine colon epithelial cells in vitro. Altern Lab Anim.

[19] Rusu D, Loret S, Peulen O, Mainil J, Dandrifosse G (2005). Immunochemical, biomolecular and biochemical characterization of bovine epithelial intestinal primocultures. BMC Cell Biol.

[20] Roodsant T, Navis M, Aknouch I, Renes IB, van Elburg RM, Pajkrt D, Wolthers KC, Schultsz C, van der Ark KCH, Sridhar A, Muncan V (2020). A human 2D primary organoid-derived epithelial monolayer model to study host-pathogen interaction in the small intestine. Front Cell Infect Microbiol.

[21] Thorne CA, Chen IW, Sanman LE, Cobb MH, Wu LF, Altschuler SJ (2018). Enteroid monolayers reveal an autonomous WNT and BMP circuit controlling intestinal epithelial growth and organization. Dev Cell.

[22] Moon C, VanDussen KL, Miyoshi H, Stappenbeck TS (2014). Development of a primary mouse intestinal epithelial cell monolayer culture system to evaluate factors that modulate IgA transcytosis. Mucosal Immunol.

[23] Noel G, Baetz NW, Staab JF, Donowitz M, Kovbasnjuk O, Pasetti MF, Zachos NC (2017). A primary human macrophage-enteroid co-culture model to investigate mucosal gut physiology and host-pathogen interactions. Sci Rep.

[24] Töpfer E, Pasotti A, Telopoulou A, Italiani P, Boraschi D, Ewart M-A, Wilde C (2019) Bovine colon organoids: from 3D bioprinting to cryopreserved multi-well screening platforms. Toxicol In Vitro 61:104606 10.1016/j.tiv.2019.104606 31344400

[25] Kozuka K, He Y, Koo-Mccoy S, Kumaraswamy P, Nie B, Shaw K, Chan P, Leadbetter M, He L, Lewis JG, Zhong Z, Charmot D, Balaa M, King AJ, Caldwell JS, Siegel M (2017). Development and characterization of a human and mouse intestinal epithelial cell monolayer platform. Stem Cell Reports.

[26] Sato T, Stange DE, Ferrante M, Vries RGJ, van Es JH, van den Brink S, van Houdt WJ, Pronk A, van Gorp J, Siersema PD, Clevers H (2011). Long-term expansion of epithelial organoids from human colon, adenoma, adenocarcinoma, and Barrett’s epithelium. Gastroenterology.

[27] Andersen CL, Jensen JL, Ørntoft TF (2004). Normalization of real-time quantitative reverse transcription-PCR data: a model-based variance estimation approach to identify genes suited for normalization, applied to bladder and colon cancer data sets. Cancer Res.

[28] Qi Z, Li Y, Zhao B, Xu C, Liu Y, Li H, Zhang B, Wang X, Yang X, Xie W, Li B, Han J-DJ, Chen Y-G (2017). BMP restricts stemness of intestinal Lgr5+ stem cells by directly suppressing their signature genes. Nat Commun.

[29] Altay G, Larrañaga E, Tosi S, Barriga FM, Batlle E, Fernández-Majada V, Martínez E (2019). Self-organized intestinal epithelial monolayers in crypt and villus-like domains show effective barrier function. Sci Rep.

[30] de Lau W, Kujala P, Schneeberger K, Middendorp S, Li VS, Barker N, Martens A, Hofhuis F, DeKoter RP, Peters PJJM, Nieuwenhuis E, Clevers H (2012). Peyer’s patch M cells derived from Lgr5+ stem cells require SpiB and are induced by RankL in cultured “miniguts”. Mol Cell Biol.

[31] Hondo T, Kanaya T, Takakura I, Watanabe H, Takahashi Y, Nagasawa Y, Terada S, Ohwada S, Watanabe K, Kitazawa H, Rose MT, Yamaguchi T, Aso H (2011). Cytokeratin 18 is a specific marker of bovine intestinal M cell. Am J Physiol Gastrointest Liver Physiol.

[32] Gebert A, Rothkötter HJ, Pabst R (1994). Cytokeratin 18 is an M-cell marker in porcine Peyer’s patches. Cell Tissue Res.

[33] Foster N, Ann Clark M, Jepson MA, Hirst BH (1998). Ulex europaeus 1 lectin targets microspheres to mouse Peyer’s patch M-cells in vivo. Vaccine.

[34] Knoop KA, Kumar N, Butler BR, Sakthivel SK, Taylor RT, Nochi T, Akiba H, Yagita H, Kiyono H, Williams IR (2009). RANKL is necessary and sufficient to initiate development of antigen-sampling M cells in the intestinal epithelium. J Immunol.

[35] Rouch JD, Scott A, Lei NY, Solorzano-Vargas RS, Wang J, Hanson EM, Kobayashi M, Lewis M, Stelzner MG, Dunn JCY, Eckmann L, Martín MG (2016) Development of functional microfold (M) cells from intestinal stem cells in primary human enteroids. PLoS One 11:e0148216 10.1371/journal.pone.0148216 PMC4731053 26820624

[36] Co JY, Margalef-Català M, Li X, Mah AT, Kuo CJ, Monack DM, Amieva MR (2019). Controlling epithelial polarity: a human enteroid model for host-pathogen interactions. Cell Rep.

[37] Smith D, Price DRG, Burrells A, Faber MN, Hildersley KA, Chintoan-Uta C, Chapuis AF, Stevens M, Stevenson K, Burgess STG, Innes EA, Nisbet AJ, McNeilly TN (2021) The development of ovine gastric and intestinal organoids for studying ruminant host-pathogen interactions. Front Cell Infect Microbiol 11:733811 10.3389/fcimb.2021.733811 PMC8456012 34568096

[38] Garcia MA, Nelson WJ, Chavez N (2018) Cell-cell junctions organize structural and signaling networks. Cold Spring Harb Perspect Biol 10:a029181 10.1101/cshperspect.a029181 PMC5773398 28600395

[39] Perrais M, Chen X, Perez-Moreno M, Gumbiner BM (2007). E-cadherin homophilic ligation inhibits cell growth and epidermal growth factor receptor signaling independently of other cell interactions. Mol Biol Cell.

[40] Srinivasan B, Kolli AR, Esch MB, Abaci HE, Shuler ML, Hickman JJ (2015). TEER measurement techniques for in vitro barrier model systems. J Lab Autom.

[41] Clevers H (2013). The intestinal crypt, a prototype stem cell compartment. Cell.

[42] Pongkorpsakol P, Satianrapapong W, Wongkrasant P, Steinhagen PR, Tuangkijkul N, Pathomthongtaweechai N, Muanprasat C (2021). Establishment of intestinal epithelial cell monolayers and their use in calcium switch assay for assessment of intestinal tight junction assembly. Methods Mol Biol.

